# Establishing the Role of Metal, Interface, and Vacancy Sites in Pt/TiO_2_-Catalyzed Acetic Acid Hydrodeoxygenation

**DOI:** 10.1021/acs.jpcc.5c00447

**Published:** 2025-04-09

**Authors:** Sean A. Tacey, Carrie A. Farberow

**Affiliations:** Catalytic Carbon Transformation & Scale Up Center, National Renewable Energy Laboratory, Golden, Colorado 80401, United States

## Abstract

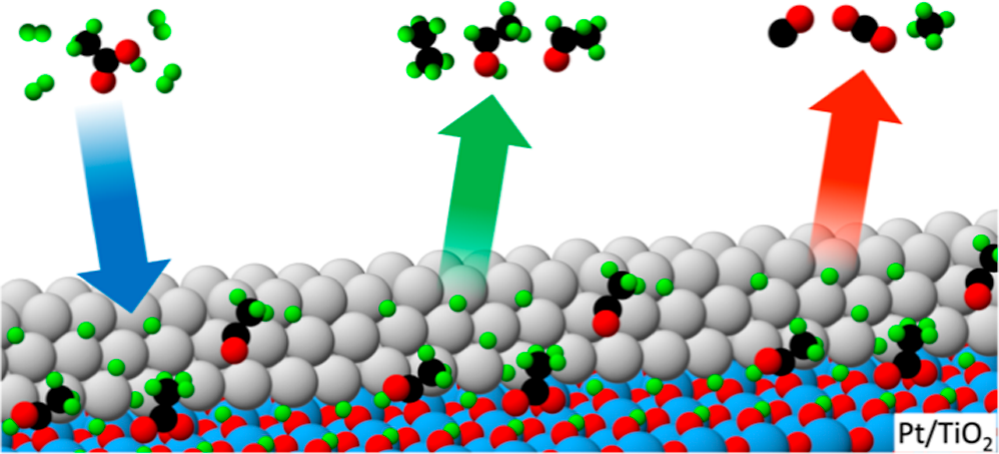

Catalytic hydrodeoxygenation (HDO) following catalytic fast pyrolysis (CFP) offers an approach to convert the vapor-phase product of biomass pyrolysis to a stable bio-oil product by reducing the oxygen content. Fundamental insights into the HDO of carboxylic acids, which are a corrosive and acidic CFP product, on promising catalyst materials, such as Pt/TiO_2_, are needed to inform the design of multifunctional HDO catalysts with improved carbon efficiency. In this contribution, density functional theory (DFT) calculations were used to assess the role of Pt-metal and Pt–TiO_2_-interface sites on acetic acid HDO (AA-HDO), and to determine the effect of interfacial oxygen vacancies at the Pt–TiO_2_ interface, by calculating the reaction energetics for key AA-HDO surface intermediates and elementary steps on each site type. Pt-metal sites, modeled via Pt(111), preferred to form undesired decarboxylation products (CH_4_ and CO_2_), whereas Pt–TiO_2_-interface sites, modeled via an anatase-supported Pt nanowire, favored the formation of desired deoxygenation products (acetaldehyde and ethane). Interfacial-vacancy sites lowered the activation energy barrier for the first C–O bond-scission step in AA-HDO, predicted to be the rate-limiting step for AA-HDO at the Pt–TiO_2_ interface in the absence of a vacancy. These atomistic insights reveal the importance of metal-metal oxide interface sites in AA-HDO selectivity and can be used to inform the rational design of improved HDO catalysts.

## 1 Introduction

As global energy demand grows, the implementation of atom- and energy-efficient processes becomes increasingly more critical. The light duty vehicle market has seen a surge of electric vehicles in recent years, but electrification of heavy duty vehicles still presents significant challenges. Alternative energy sources, like liquid biofuels, can provide a cost-effective option for meeting some of these higher energy density needs.^1−3^ Catalytic fast pyrolysis (CFP), in which solid biomass is fed to a fast pyrolysis unit run at moderate temperatures to produce a vapor mixture of oxygenated hydrocarbons which are catalytically upgraded, is a promising, and thus widely explored, route to produce liquid biofuels from solid biomass feedstocks.^4−8^ The high oxygen content of the CFP vapor mixture necessitates hydrodeoxygenation (HDO) reactions, either during (in situ) or following (ex situ) CFP, to reduce the oxygen content and create a more stable bio-oil product. This bio-oil can undergo further processing, e.g., hydrotreating, to meet required specifications for liquid transportation fuels.

Zeolite catalysts have historically been used to upgrade CFP vapors, but suffer from low carbon efficiency due to cracking side reactions that form undesired C1 products (i.e., CH_4_, CO, and CO_2_) and coke precursors that deactivate the catalyst.^9−14^ Bifunctional metal-acid catalysts are another promising class of materials for HDO catalysis, as they can reduce the oxygen content of the CFP vapor while mitigating the formation of C1 products.^15,16^ Recent advances on bifunctional metal-acid catalysts have shown metal-carbide,^17−19^ metal-phosphide,^20−24^ and metal-nitride materials^25−27^ as promising catalysts for HDO. Noble metals supported on reducible metal oxides (e.g., Pt/TiO_2_), which are also effective HDO catalysts and are widely commercially available, have also been the subject of numerous HDO studies, including HDO of model compounds and whole CFP vapor mixtures.^16,28−32^

In the case of reducible metal oxide-supported noble-metal catalysts, model-compound studies have largely focused on determining the attributes that enable the selective production of desired deoxygenation products over undesired decarbonylation/decarboxylation products that reduce the overall carbon efficiency. Prior reports have primarily evaluated phenolic compounds, including phenol,^33,34^ guaiacol,^35−40^ cresol,^8,41−45^ and anisole,^46,47^ because these tend to be particularly recalcitrant compounds. Griffin and co-workers showed that, for *m*-cresol HDO, supporting Pt on TiO_2_ increases the HDO activity relative to Pt/C, and the carbon selectivity toward the desired deoxygenation product, toluene, increased from ca. 45% for Pt/C to ca. 80% for Pt/TiO_2_.^8^ Density functional theory (DFT) calculations further indicated that *m*-cresol undergoes ring hydrogenation on Pt(111) to form undesired 3-methylcyclohexanone and 3-methylcyclohexanol, while anatase-TiO_2_(101) favors the formation of toluene through tautomerization and direct deoxygenation steps, suggesting a hydrogen-spillover mechanism for Pt/TiO_2_. Nelson et al. employed a combination of isotopic labeling experiments and DFT calculations to elucidate the mechanism for phenol HDO on Ru/TiO_2_.^34^ They concluded that the Ru–TiO_2_ interface promotes the desired deoxygenation pathway, where interfacial hydroxyl vacancies and interfacial water both significantly lower the barrier for C–O bond-cleavage steps. A follow-up study on *m*-cresol HDO on Ru/TiO_2_ supported these findings by demonstrating that the activation energy barrier for C–O bond cleavage was lower at the Ru–TiO_2_ interface than at Ru-metal sites, and that the presence of water further lowered the activation energy barrier for C–O bond cleavage.^41^

Carboxylic acid compounds are another major component of the CFP vapor mixture, constituting ca. 50% of the aqueous stream from CFP on a dry weight basis.^48^ The presence of carboxylic acids increases the acidity and corrosivity of the raw CFP bio-oil,^49^ necessitating their removal, preferably via chemical transformations to value-added products over the HDO catalyst to improve both the carbon efficiency and quality of the final bio-oil product. Acetic acid (AA) is the simplest C_2+_ carboxylic acid and is also a primary acidic compound in the aqueous stream from CFP. Therefore, understanding the reactivity of acetic acid on the HDO catalyst is important to determine: (i) the activity and selectivity of the catalyst for carboxylic acid deoxygenation, and (ii) how the HDO of carboxylic acids may affect catalyst lifetime and overall carbon selectivity for biomass upgrading through CFP. For example, carboxylic acid decomposition can result in the formation of catalyst poisons (e.g., CO) that diminish the activity and selectivity of the catalyst over time.^49^ Contreras-Mora and co-workers evaluated AA-HDO on carbon-supported noble-metal catalysts (Pt, Pd, Rh, Ru) and found that all catalysts preferentially formed undesired decarbonylation or decarboxylation products (CO, CO_2_, and CH_4_).^50^ He and Wang investigated the role of support identity on Pt-catalyzed AA-HDO and found that Pt/C selectively formed undesired decarbonylation products, but that Pt/TiO_2_ selectively formed desired partial and complete deoxygenation products, acetaldehyde and ethane, respectively.^49^ The higher selectivity of Pt/TiO_2_ toward acetaldehyde and ethane relative to undesired decarbonylation or decarboxylation products was attributed to the presence of vacancy sites, which were postulated to promote the reverse Mars-van Krevelen reaction of acetic acid to form acetaldehyde.^49,51^ However, undesired decarboxylation products were also formed during AA-HDO on Pt/TiO_2_.

Mechanistic insights into the role of the metal, support, and metal-metal oxide interface in Pt/TiO_2_ for HDO of the diversity of oxygenates in the CFP vapor mixture is key to informing the development of more active and selective metal-oxide-supported transition metal HDO catalysts. To advance such understanding for carboxylic acids, herein DFT calculations are performed to determine the energetically preferred reaction pathway for AA-HDO on Pt/TiO_2_. These results provide atomic-scale insights into the intrinsic activity of Pt-metal, Pt–TiO_2_-interface, and interfacial-vacancy sites in AA-HDO. In particular, the propensity of each site type to promote desired C–O bond-breaking steps versus undesired C–C bond-breaking steps during AA-HDO is evaluated and discussed.

## 2 Methods

DFT calculations were performed using the Vienna Ab initio Simulation Package (VASP).^52,53^ Exchange-correlation effects were captured using the generalized gradient approximation (GGA) through the Perdew-Burke-Ernzerhof (PBE) exchange-correlation functional.^54^ Dispersion corrections were added to the DFT calculations via the DFT-D3 method developed by Grimme and co-workers.^55^ Electron-ion interactions were described using projector augmented-wave (PAW) potentials with valence-electron wave functions expanded using a plane-wave basis set with a cutoff energy of 500 eV.^56,57^

**Figure 1:**
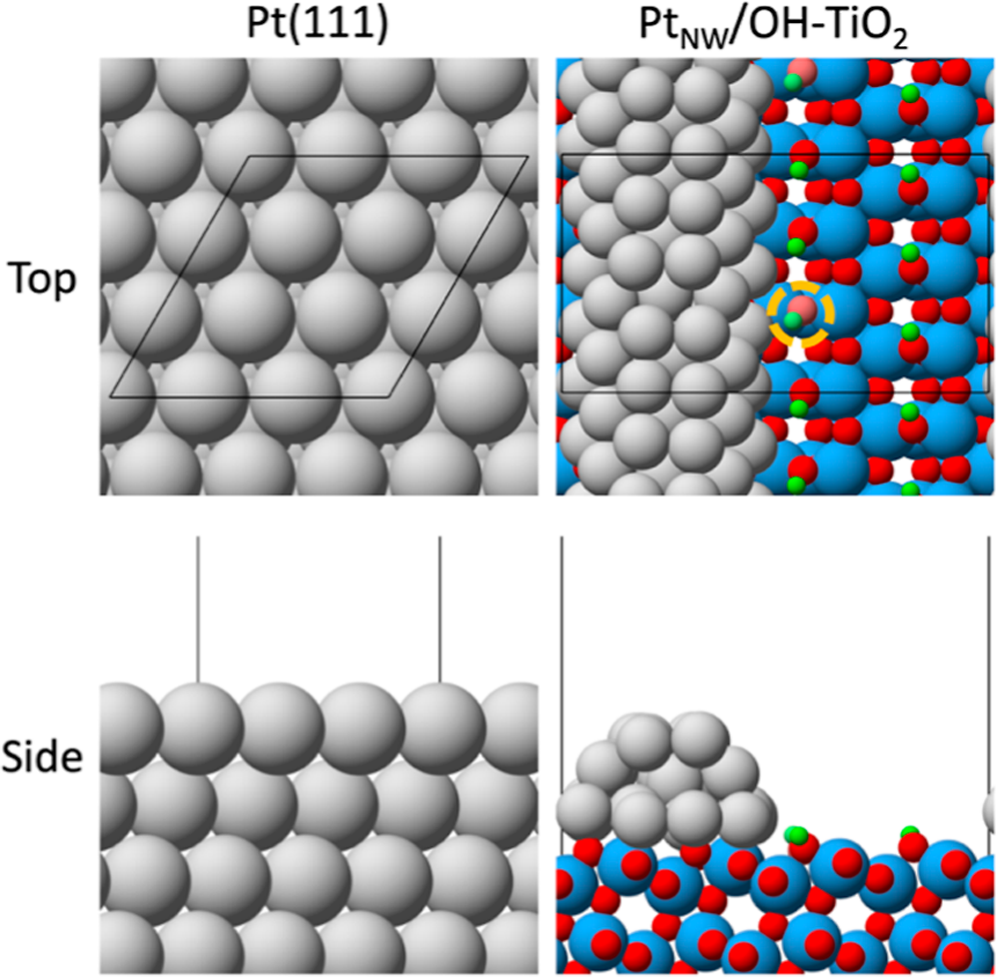
Top and side views of Pt(111) and Pt_NW_/OH-TiO_2_. The light-shaded OH group outlined by the dashed-yellow circle in the Pt_NW_/OH_v_-TiO_2_ model indicates the OH removed to form an interfacial vacancy (Pt_NW_/OH_v_-TiO_2_). Black lines denote the surface unit cell. Atom colors: Pt—gray, H—green, O—red, and Ti—blue.

Pt-metal sites were modeled using a Pt(111) slab with a (3 × 3) periodic surface unit cell repeated in a supercell geometry (Figure 1). The Pt(111) slab consisted of four layers; the bottom two layers were fixed in their bulk-truncated positions, while the top two layers and all adsorbates could freely relax. The DFT-calculated lattice constant for Pt was 3.920 Å, in close agreement with the experimental value of 3.924 Å.^58^ A 4 × 4 × 1 Monkhorst–Pack *k*-point mesh was used to sample the surface Brillouin zone of Pt(111).^59^ Pt–TiO_2_-interface sites were modeled through an anatase-supported Pt-nanowire model. Anatase was chosen due to its high abundance (ca. 70–80%^60^) in the P25 support widely used in prior investigations of HDO on TiO_2_-supported Pt catalysts.^8,16,38,61^ Due to its thermodynamic stability,^62^ the (101) facet of anatase TiO_2_ was used to simulate the TiO_2_ surface via a (4 × 3) anatase-TiO_2_(101) periodic surface unit cell (aTiO_2_(101)). DFT-calculated lattice constants for anatase were 3.865 and 9.627 Å for *a* and *c*, respectively, in agreement with the experimental values of 3.785 and 9.514 Å.^58^ The supported Pt nanowire was developed using the lattice-matching code developed by Greeley et al.^63,64^ A three-layer Pt(100) slab was first optimized on the aTiO_2_(101) surface, followed by cleavage of the Pt(100) slab to form a Pt nanowire, exposing (100) and (111) surfaces on the top and sides of the nanowire, respectively (Figure 1). The aTiO_2_(101) slab had 12 atomic layers; the bottom six layers were fixed in their respective bulk-truncated positions, while the top six layers, the Pt nanowire, and all adsorbates could freely relax. A 1 × 2 × 1 Monkhorst–Pack *k*-point mesh was used to sample the surface Brillouin zone of the anatase-supported Pt-nanowire model.^59^ To reflect the reducing conditions in HDO chemistry, surface-O anions on the TiO_2_(101) support were terminated with H atoms to form surface-OH groups. The anatase-supported Pt-nanowire model is referred to herein as “Pt_NW_/OH-TiO_2_.” The role of an interfacial-OH vacancy was investigated by removing one interfacial-OH group [“Pt_NW_/OH_v_-TiO_2_”; (Figure 1)].

For all surface models, a vacuum layer at least 10 Å thick separated successive slabs in the *z* direction. Adsorption was limited to one of the two exposed surfaces with adjustments made to the electrostatic potential to account for the adsorbate-induced dipole.^65,66^ All structures were relaxed until the Hellmann–Feynman forces acting upon each atom fell below 0.02 eV/Å. Spin-polarized adjustments were made to the GGA for Pt_NW_/OH-TiO_2_ and Pt_NW_/OH_v_-TiO_2_; no such adjustment was made for Pt(111). The +*U* correction was implemented to account for on-site Coulombic repulsions in anatase following the approach of Dudarev and co-workers^67^ with a *U*_eff_ of 2.5 eV.^8,68^

The interaction between surface intermediates and surface models was quantified using the binding energy (*E*_B_), defined as
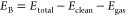
1where *E*_total_, *E*_clean_, and *E*_gas_ are the total energy of the adsorbate + surface complex, the clean surface, and the gas-phase adsorbate, respectively. Activation energy barriers were determined using climbing-image nudged elastic band (CI-NEB) calculations, in which seven images were linearly interpolated between the initial and final state.^69,70^ CI-NEB calculations converged once the maximum force acting upon each atom fell below 0.05 eV/Å for each image. Transition states were verified by observing a single imaginary vibrational frequency in the transition-state structure.^71^ Vibrational frequency analyses were performed by fixing all surface atoms and relaxing only bound surface-intermediate atoms. Bonds were oscillated using a step size of 0.015 Å. The activation energy barrier (*E*_a_) for each elementary step was calculated as the energy difference between the transition (*E*_TS_) and most stable initial (*E*_MS-IS_) states; i.e.
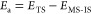
2

## 3 Results and Discussion

**Scheme 1:**
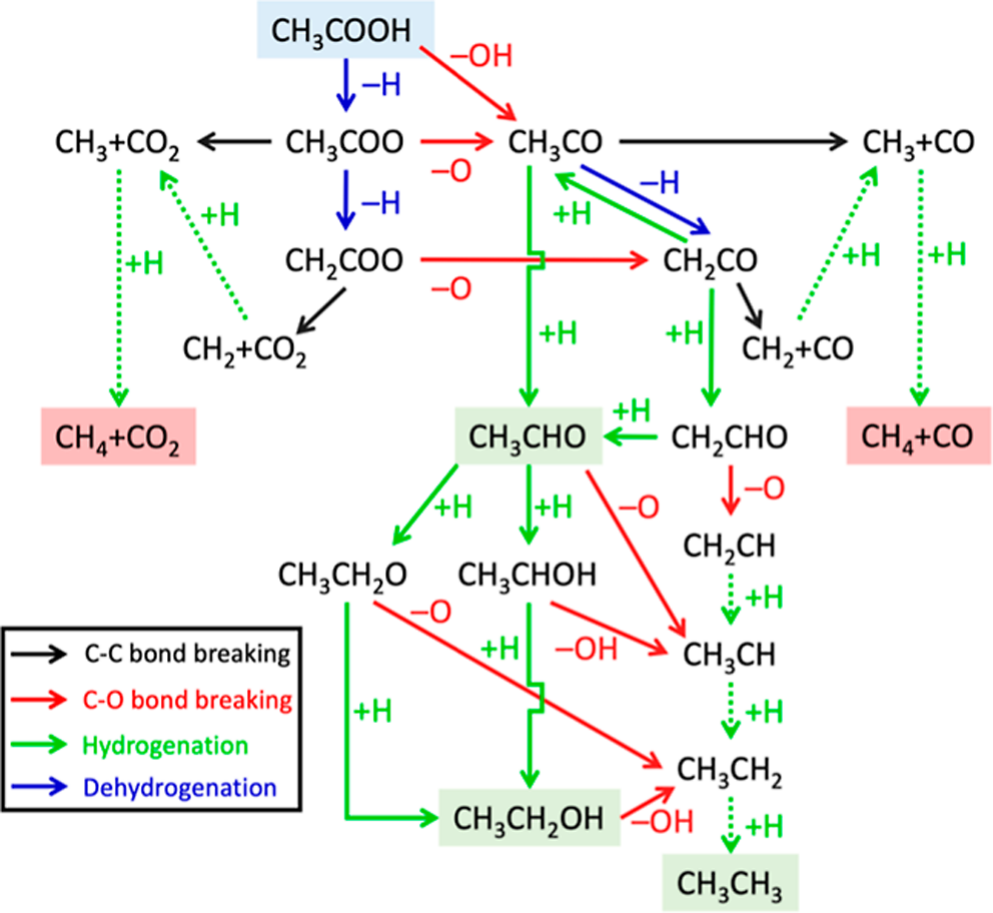
Reaction Pathways for AA-HDO Including C–C Bond-Dissociation (Black Arrows), C–O Bond-Dissociation (Red Arrows), Dehydrogenation (Blue Arrows), and Hydrogenation (Green Arrows) Elementary Steps Blue, green, and red shading indicates reactants, desired products, and undesired products, respectively. Elementary hydrogenation steps that follow the second C–O or a C–C bond-breaking step were not explicitly calculated (dashed green arrows).

At typical HDO temperatures and pressures between 423–573 K and 1–4 bar, respectively, the primary AA-HDO products observed experimentally include acetaldehyde, ethane, and ethanol, while CO, CO_2_, and methane are the undesired decarbonylation/decarboxylation products.^49,72,73^ In a previous report utilizing a combination of temperature-programmed desorption (TPD), temperature-programmed reaction (TPR), and diffuse reflectance infrared Fourier transform spectroscopy (DRIFTS) measurements, Rachmady and Vannice determined that acetate and acyl species (e.g., CH_2_CO and CH_3_CO) are formed as surface-bound intermediates during AA-HDO on Pt/TiO_2_ catalysts.^73^ Based on these prior experimental observations, postulated elementary steps and corresponding intermediates involved in AA-HDO reaction pathways were defined (Scheme 1). We note that isopropyl alcohol and ethyl acetate formation have also been observed as stable gas-phase products in experiments at lower abundance than ethane and acetaldehyde;^49,73^ C–C bond-formation steps and the corresponding products are excluded from the present study for computational tractability and, thus, pathways to form these more minor products are beyond the scope of the present work. Activation energy barriers for hydrogenation steps following the C–C or second C–O bond-breaking steps, which are likely kinetically less important given the relative barriers computed for hydrogenation elementary steps earlier in the reaction pathways, were also not calculated to simplify the analysis. Furthermore, because this study focused on assessing the intrinsic activity of Pt-metal, Pt–TiO_2_-interface, and interfacial-vacancy sites in AA-HDO, entropic contributions to the stability and reactivity of the studied AA-HDO surface intermediates are not included. Collectively, these assumptions and prior findings informed the relevant surface intermediates, products, and elementary steps considered here; the resulting hypothesized AA-HDO reaction pathways are depicted in Scheme 1.

### 3.1 AA-HDO Surface-Intermediate Adsorption

**Figure 2:**
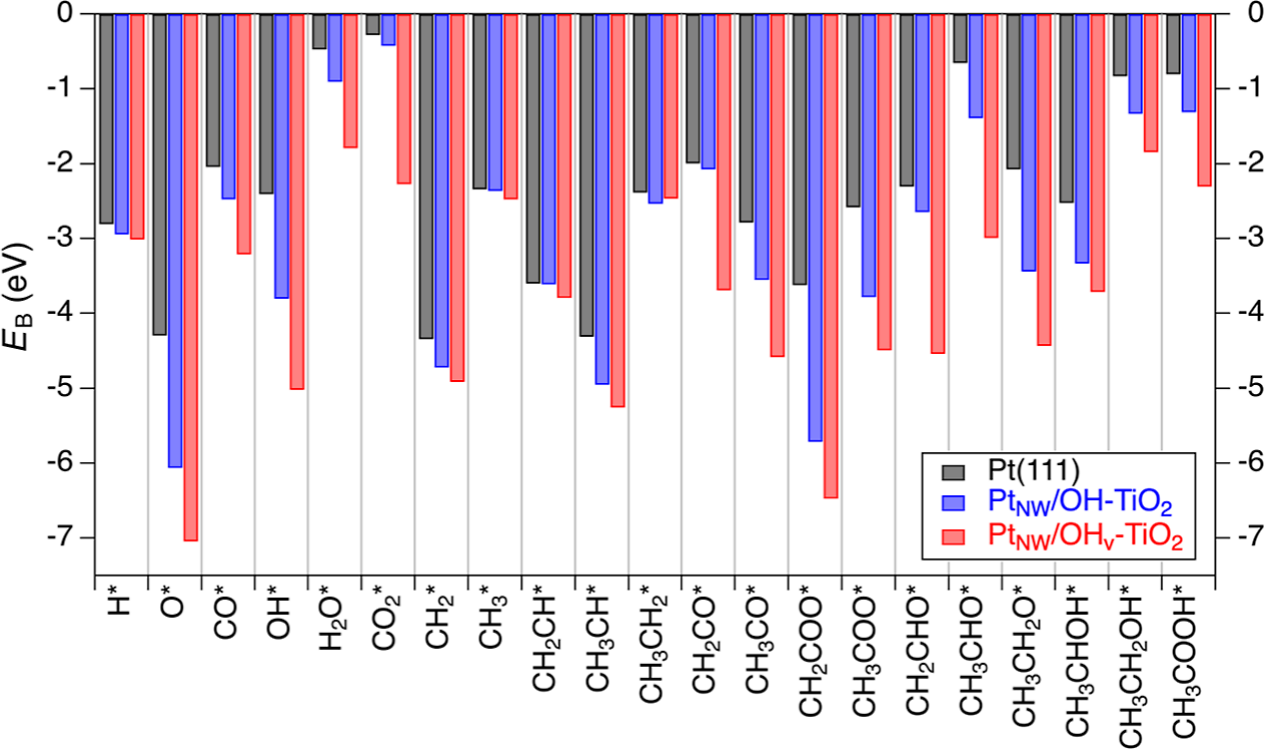
Adsorption energies (*E*_B_, in eV; 1 eV = 96.5 kJ/mol) for surface intermediates on Pt(111) (black bars), Pt_NW_/OH-TiO_2_ (blue bars), and Pt_NW_/OH_v_-TiO_2_ (red bars). * denotes an adsorbed species.

Adsorption energetics were calculated for all surface intermediates in the AA-HDO reaction mechanism (Scheme 1) on Pt(111), Pt_NW_/OH-TiO_2_, and Pt_NW_/OH_v_-TiO_2_ (Figure 2). The optimized configurations of each intermediate on each of the three surface models are provided in Figures S1–S3. On Pt(111), the strongest binding intermediate is CH_2_* (*E*_B(Pt(111))_ = −4.34 eV) and the weakest binding species is CO_2_* (*E*_B(Pt(111))_ = −0.28 eV). The Pt–TiO_2_ interface results in stronger adsorption of each intermediate relative to Pt(111) by an average of 0.67 eV, with adsorption energies on Pt_NW_/OH-TiO_2_ ranging from −6.06 eV for O* to −0.42 eV for CO_2_*. Relative to Pt(111), CH_2_CH* (−0.01 eV) and CH_3_* (−0.02 eV) are least stabilized on Pt_NW_/OH-TiO_2_, while CH_2_COO* is most stabilized (−2.09 eV). Due to increased interactions between carbonyl and/or hydroxyl groups with the interface (e.g., H-bonding interactions with surface hydroxyls on TiO_2_), oxygen-containing intermediates were more stabilized on Pt_NW_/OH-TiO_2_ relative to Pt(111) (−0.85 eV on average) than nonoxygen-containing surface intermediates (−0.22 eV on average).

To form an interfacial vacancy to produce Pt_NW_/OH_v_-TiO_2_ from Pt_NW_/OH-TiO_2_, three steps are required: (i) H_2_ activation to form 2H* on the Pt metal; (ii) H* spillover from Pt to an interfacial OH to form an interfacial H_2_O; and (iii) desorption of H_2_O from the interface.^34,74,75^ H_2_ activation on Pt readily occurs,^76^ whereas the reaction energy for H* spillover and interfacial-H_2_O desorption were calculated as 0.80 and 1.76 eV, respectively. At typical AA-HDO reaction temperatures of 200–300 °C,^49,77^ ca. 1.3–2.0 eV is available assuming a Boltzmann distribution,^78^ indicating that interfacial vacancies can form under the higher temperature range for typical AA-HDO reaction conditions. In the presence of an interfacial OH vacancy, each AA-HDO intermediate binds stronger than on Pt(111) and Pt_NW_/OH-TiO_2_ by an average of 1.56 and 0.84 eV, respectively, except for CH_3_CH_2_* which binds slightly weaker to Pt_NW_/OH_v_-TiO_2_ compared to Pt_NW_/OH-TiO_2_ (−2.46 eV vs −2.53 eV, respectively). Adsorption energies for surface intermediates on Pt_NW_/OH_v_-TiO_2_ vary from −7.04 eV for O* to −1.79 eV for H_2_O*. The O-containing surface intermediates universally bind to the vacancy site through an O atom, whereas the remaining surface intermediates, aside from CH_3_*, do not bind to the vacancy site and instead bind to interfacial-Pt sites (Figure S3). As such, similar to adsorption energies for surface intermediates on Pt_NW_/OH-TiO_2_ relative to Pt(111), there is a larger average stabilization of O-containing intermediates (1.92 eV) on Pt_NW_/OH_v_-TiO_2_ as compared to non-O-containing intermediates (0.35 eV) when comparing against binding energies on Pt(111). Based on this initial observation, one could speculate that interfacial vacancies may reduce reaction energies and activation energy barriers for C–O bond-breaking steps to a greater extent than C–C bond-breaking steps.

### 3.2 Reaction Energetics and Pathways for AA-HDO

**Figure 3:**
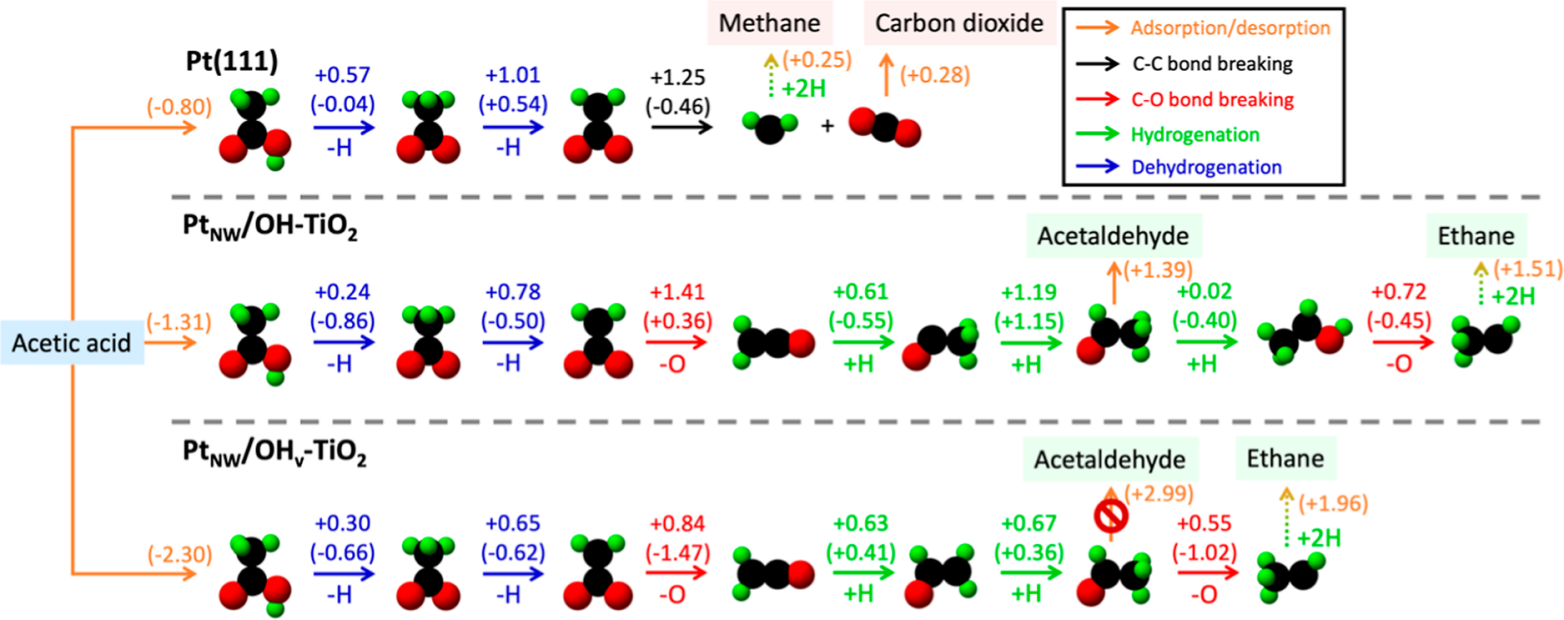
Minimum-energy pathway for AA-HDO on Pt(111), Pt_NW_/OH-TiO_2_, and Pt_NW_/OH_v_-TiO_2_. Species molecule snapshots and text names represent adsorbed and gas-phase species, respectively. Values next to arrows denote activation energy barriers (reaction energies, in eV). Dashed arrows represent subsequent elementary hydrogenation steps for product formation that were not explicitly evaluated. Species text shading indicate reactants (blue), desired HDO products (green), and undesired HDO products (red).

**Figure 4:**
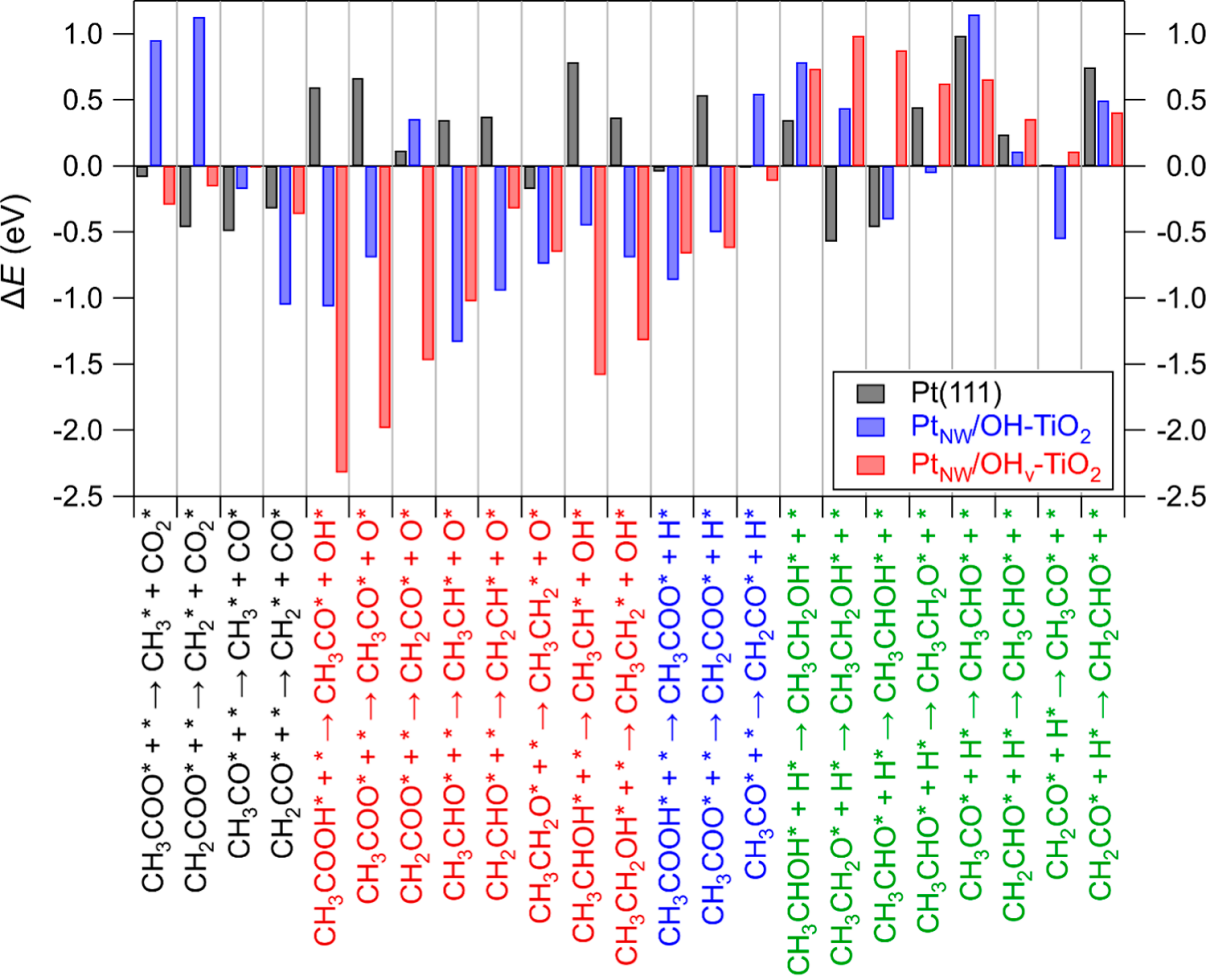
Reaction energies (Δ*E*, in eV) for C–C bond-dissociation (black text), C–O bond-dissociation (red text), dehydrogenation (blue text), and hydrogenation (green text) elementary steps on Pt(111) (black bars), Pt_NW_/OH-TiO_2_ (blue bars), and Pt_NW_/OH_v_-TiO_2_ (red bars). * denotes an adsorbed species or vacant site.

**Figure 5:**
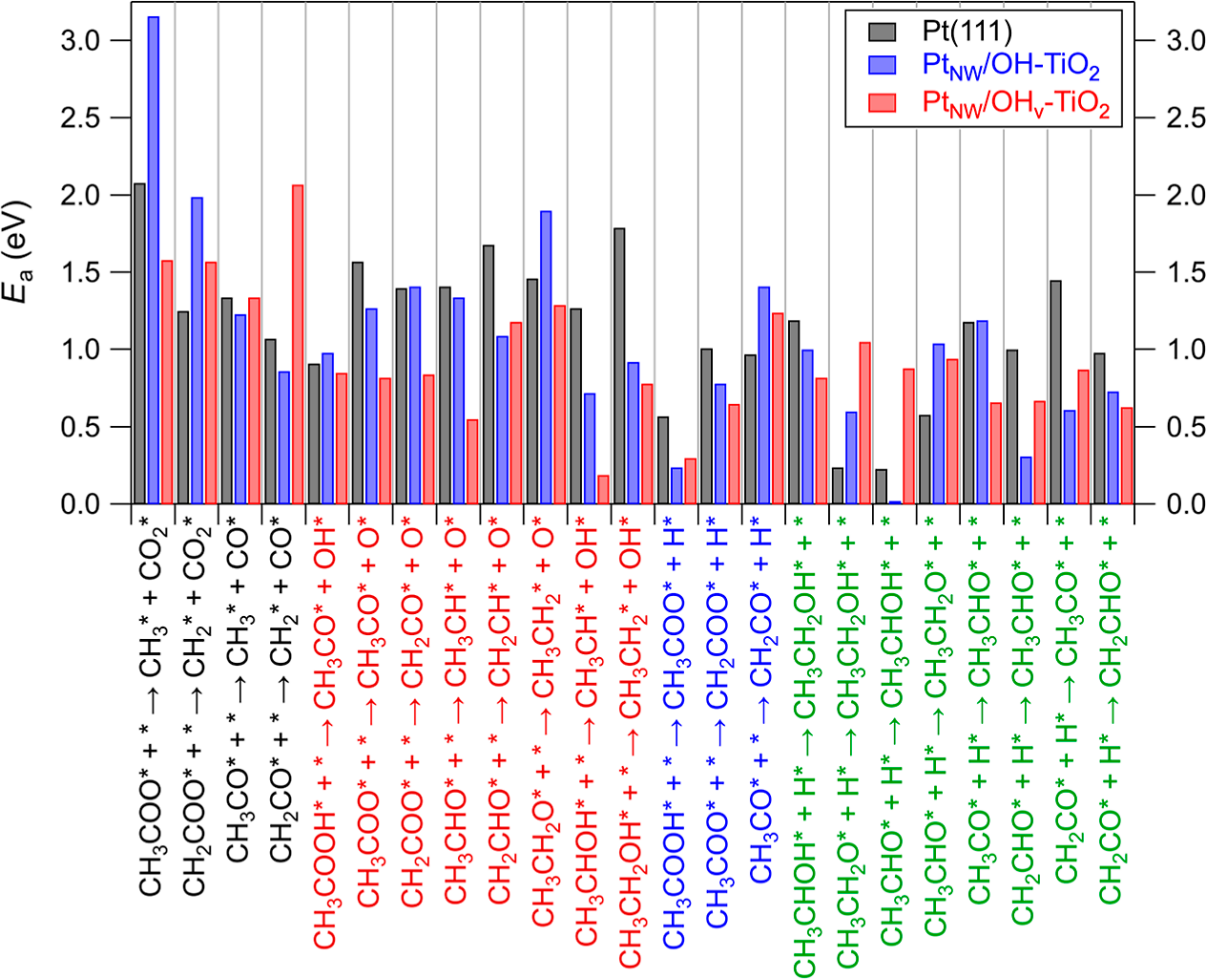
Activation energy barriers (*E*_a_, in eV) for C–C bond-dissociation (black text), C–O bond-dissociation (red text), dehydrogenation (blue text), and hydrogenation (green text) elementary steps on Pt(111) (black bars), Pt_NW_/OH-TiO_2_ (blue bars), and Pt_NW_/OH_v_-TiO_2_ (red bars). * denotes an adsorbed species or vacant site.

To further evaluate whether, relative to Pt-metal sites, Pt–TiO_2_-interface sites are predicted to shift the AA-HDO selectivity toward desired deoxygenation products, the activation energy barriers for each step in the AA-HDO pathway (Scheme 1) were utilized to construct the minimum energy pathways (MEPs) on each surface model assuming the reaction proceeds via the minimum activation barrier step at each branch point in the reaction network beginning with adsorbed acetic acid (Figures 3 and S4–S6). The reaction energetics and activation energy barriers used to generate the MEPs for Pt(111), Pt_NW_/OH-TiO_2_, and Pt_NW_/OH_v_-TiO_2_ (Scheme 1) are summarized in Figures 4 and 5, respectively (tabulated in Table S1).

On Pt(111), AA-HDO is predicted to proceed first through two sequential dehydrogenation steps to form CH_3_COO* then CH_2_COO*, followed by C–C bond breaking and subsequent hydrogenation to form the decarboxylation products, CH_4_ and CO_2_. However, previous studies found that Pt/C catalysts primarily form decarbonylation products, CH_4_ and CO.^49,50^ This discrepancy may be due to the ability of Pt/C to catalyze the reverse water-gas shift (RWGS) reaction,^79^ thereby converting the CO_2_ product to CO. Alternatively, because CH_3_COO* + H* is an energetic minima for the MEP of AA-HDO on Pt(111) (Figure S4), CH_3_COO* may be an abundant surface intermediate on Pt/C during AA-HDO. However, effects of lateral interactions with high-coverage adsorbates on the simulated reaction energetics are beyond the scope of the present study. Evaluation of the role of these spectator species on the predicted AA-HDO reaction pathway would be a valuable future contribution. Yet, the calculated results for the low-coverage Pt(111) model system do correctly predict the formation of undesired C1 products, indicating the propensity for Pt-metal sites to catalyze C–C bond-breaking to ultimately form undesired AA-HDO products that reduce the overall HDO carbon efficiency.

On Pt_NW_/OH-TiO_2_, AA-HDO is also predicted to proceed through sequential acetic acid dehydrogenation to CH_3_COO* and then to CH_2_COO* (Figure 3). However, rather than undergoing C–C bond cleavage as on Pt(111), CH_2_COO* undergoes C–O bond cleavage to form the acyl species, CH_2_CO*. Next, acetaldehyde is formed via two subsequent hydrogenation steps, which proceed via CH_3_CO* followed by CH_3_CHO*. Hydrogenation of surface-bound acetaldehyde to CH_3_CHOH* (0.02 eV) is energetically favored over the desorption of acetaldehyde (1.39 eV). Once CH_3_CHOH* is formed, it preferentially undergoes deoxygenation to CH_3_CH*, after which subsequent hydrogenation and desorption steps result in the formation of the fully deoxygenated gas-phase product ethane. The formation of adsorbed ethanol from CH_3_CHOH* is unfavorable relative to CH_3_CH* formation, which proceed via activation energy barriers of 1.00 and 0.72 eV, respectively. While ethane is the primary product predicted by following the MEP on Pt_NW_/OH-TiO_2_, CH_3_CH* hydrogenation to form gas-phase ethane is endothermic by 1.51 eV; therefore, the energetics of OH* removal as H_2_O may play an important role in determining the reversibility of the elementary step CH_3_CHOH* + * → CH_3_CH* + OH*, and thus the selectivity between acetaldehyde and ethane on Pt_NW_/OH-TiO_2_ (Figure S5). Regardless, the formation of the desired AA-HDO deoxygenation products, ethane and acetaldehyde, is predicted to occur on Pt_NW_/OH-TiO_2_, while ethanol formation is not predicted, in agreement with previous experimental observations by He and Wang.^49^

In the presence of an interfacial-OH vacancy, the MEP largely follows the same pathway as predicted for Pt_NW_/OH-TiO_2_ in the absence of an OH vacancy (Figure 3), although there are several notable differences. The activation energy barrier for the first C–O bond-dissociation step (CH_2_COO* + * → CH_2_CO* + O*) on Pt_NW_/OH_v_-TiO_2_ (0.84 eV) is notably lower than that on Pt_NW_/OH-TiO_2_ (1.41 eV), which is likely a result of the stronger binding of the oxygenated intermediate at the vacancy site. A more minor difference in the predicted pathway is the order of CH_2_CO* hydrogenation steps to produce acetaldehyde. On Pt_NW_/OH-TiO_2_, CH_2_CO* undergoes hydrogenation first at the α-carbon atom to form CH_3_CO*, whereas on Pt_NW_/OH_v_-TiO_2_ CH_2_CO* undergoes hydrogenation first at the carbonyl carbon to form CH_2_CHO*. Another, perhaps more impactful distinction between the AA-HDO energetics calculated in the absence and presence of an interfacial vacancy involves the desorption energy of the acetaldehyde product. On Pt_NW_/OH-TiO_2_ the energy to desorb acetaldehyde is 1.39 eV, whereas when acetaldehyde is bound at an interfacial-vacancy site the desorption energy is prohibitively high at 2.99 eV due to the strong interaction between the O in the acetaldehyde and the neighboring Ti sites. Acetaldehyde bound at an interfacial-OH vacancy site is thus more likely to undergo C–O bond-breaking to form ethane (*E*_a_ = 0.55 eV), preferentially healing the vacancy rather than desorbing directly to form CH_3_CHO_(g)_. Considered alongside the aforementioned reduced barrier for C–O bond-breaking in CH_2_COO*, these results demonstrate that interfacial vacancies likely facilitate C–O bond-scission steps in AA-HDO, and thus interfacial vacancy concentration may play a key role in the AA-HDO deoxygenation product distribution (acetaldehyde versus ethane). These results are well-aligned with prior experimental findings that, in the absence of clear atomistic evidence, speculated that higher AA-HDO selectivities toward acetaldehyde and ethane could be attributed to the presence of vacancy sites in the metal-oxide support that catalyzed C–O bond breaking steps via a reverse Mars-van Krevelen mechanism.^49,51^ Yet, due to the relatively high barrier associated with interfacial-vacancy formation (1.76 eV), the formation of the vacancy sites could be a key rate-controlling step for vacancy-mediated AA-HDO on TiO_2_-supported Pt catalysts.

For Pt(111), C–C and C–O bond-breaking steps are, on average, exothermic by −0.34 eV and endothermic by +0.39 eV, respectively, supporting the aforementioned predictions for preferential formation of undesired C1 products on this surface (Figure 4, Table S1). The average values for C–C and C–O bond breaking step activation energy barriers on Pt(111) are equivalent at 1.44 eV. For Pt_NW_/OH-TiO_2_, the reaction energy trends instead indicate that C–C bond-breaking steps are endothermic on average (+0.22 eV), whereas C–O bond-breaking steps are on average exothermic (−0.69 eV). In this case, these trends in the reaction energies are further reflected by the average activation energy barriers, as C–O bond-breaking steps on average have lower activation barriers than C–C bond-breaking steps (1.22 eV vs 1.81 eV). Furthermore, C–O bond-breaking steps have lower activation energy barriers on Pt_NW_/OH-TiO_2_ compared to Pt(111) by an average of 0.23 eV (Figure 5, Table S1). These results clearly indicate the important role of the TiO_2_ support in enhancing selectivity toward deoxygenation products. In the case of Pt_NW_/OH_v_-TiO_2_, as compared to both Pt(111) and Pt_NW_/OH-TiO_2_, C–O bond-breaking steps in the presence of an interfacial vacancy were most exothermic (−1.33 eV) and had the lowest average activation energy barriers (0.85 eV). C–C bond-breaking steps on Pt_NW_/OH_v_-TiO_2_ have average reaction energies (−0.20 eV) and activation energy barriers (1.64 eV) between those for Pt(111) and Pt_NW_/OH-TiO_2_.

For both the Pt_NW_/OH-TiO_2_ and Pt_NW_/OH_v_-TiO_2_ surface models, H atoms in the TiO_2_ surface-OH groups, denoted as [OH]_s_, were observed to participate in some energetically favorable elementary hydrogenation reaction steps in either a single-step (Figure S5; e.g., in CH_3_CHO* + [OH]_s_ → CH_3_CHOH* + [O]_s_ on Pt_NW_/OH-TiO_2_) or multistep (Figures S5 and S6, e.g., in CH_2_CHO* + [OH]_s_ → CH_2_CHO* + H* + [O]_s_ → CH_3_CHO* + [O]_s_ on Pt_NW_/OH_v_-TiO_2_) pathway. In both cases, the hydrogen atom in the [OH]_s_ species is consumed in the hydrogenation reaction, leaving a [O]_s_ surface species. The [OH]_s_ group is then regenerated by an H* atom (i.e., [O]_s_ + H* → [OH]_s_). These observations highlight the important and varying mechanistic roles that TiO_2_ surface OH groups and oxygen vacancies can play in HDO surface chemistry.

Based on the predicted MEPs for Pt_NW_/OH-TiO_2_ and Pt_NW_/OH_v_-TiO_2_ (Figures 3, S4, and S5), both acetate and acyl species (CH_2_CO*, CH_3_CO*) are predicted intermediates during AA-HDO regardless of whether the reaction proceeds via a vacancy site. This finding is consistent with Rachmady and Vannice who observed the formation of both surface-bound acetate and acyl species during AA-HDO using a combination of DRIFTS, TPD, and TPR measurements.^73^ They suggested, however, that acetate is a spectator species based on the decomposition temperature of each surface intermediate, while the results reported here indicate that both species are active intermediates in the AA-HDO pathway. Notably, the first C–O bond-scission step, which involves acetate, (CH_2_COO* + * → CH_2_CO* + O*) exhibits the highest barrier in the predicted minimum-energy pathway for AA-HDO on Pt_NW_/OH-TiO_2_, and could therefore be an important rate-controlling step for AA-HDO at Pt-TiO_2_ interface sites; thus, the results reported herein are consistent with the role of acetate as an abundant surface intermediate during the reaction. Further analyses could be helpful to determine the effect of abundant acetate and/or acyl species on the calculated reaction energetics and corresponding pathways for AA-HDO. For vacancy-mediated AA-HDO on TiO_2_-supported Pt catalysts, vacancy regeneration and product desorption are other likely key rate-controlling steps. Future combined experimental and theoretical investigations, leveraging the results reported here, would be valuable to more fully illustrate the atomic-scale roles of the various possible site types, including cooperative pathways that may leverage the site heterogeneity, the impact of temperature and entropic effects on the predicted pathways for AA-HDO, and the role of vacancy formation, healing, and regeneration on the observed AA-HDO reaction rates and product selectivities.

## 4 Conclusions

DFT calculations were employed to evaluate the role of Pt-metal, Pt–TiO_2_-interface, and interfacial hydroxyl vacancy sites in AA-HDO. Pt–TiO_2_-interface and interfacial-vacancy sites, simulated through an anatase-TiO_2_(101)-supported Pt-nanowire model, were observed to bind AA-HDO surface intermediates stronger than Pt-metal sites [simulated via a Pt(111) surface slab]. Pt–TiO_2_ interface and interfacial-vacancy sites exhibited increased thermodynamic and kinetic favorability toward C–O bond-dissociation steps as compared to Pt-metal sites. Based on the predicted MEPs for AA-HDO on each model system, Pt-metal sites favored the formation of undesired decarboxylation products, whereas Pt–TiO_2_-interface sites, irrespective of the presence of an interfacial vacancy, favored the formation of the desired deoxygenation products, acetaldehyde and ethane. Overall, the results from this analysis indicate that (i) metal-metal oxide interface sites are likely required to promote the formation of desired deoxygenation products from AA-HDO on supported Pt catalysts; (ii) interfacial vacancies may facilitate C–O bond-breaking steps by further reducing activation energy barriers; and (iii) the concentration of interfacial-vacancy sites may play an important role in dictating the product selectivity to partially versus fully deoxygenated products (e.g., ethane versus acetaldehyde in AA-HDO) on Pt/TiO_2_ catalysts.

Ultimately, these findings indicate that increasing concentrations of interface sites can facilitate deoxygenation over decarbonylation/decarboxylation product formation which can inform design strategies for improved catalysts for carboxylic acid HDO. For example, smaller catalyst particles could be utilized to increase the concentration of interface sites. In addition, strategies such as the use of atomic-layer deposition (ALD) to deposit thin, partial TiO_2_ coatings on Pt catalysts, leading to the blocking of metal sites which favor undesired C1 product formation, as well as the creation of additional Pt–TiO_2_ interface sites, may prove useful. Such an approach, involving TiO_2_-overcoated Pd and Pt catalysts, has shown promise for improving the activity and selectivity for aromatic hydrogenation^80^ and ketone HDO chemistries;^75^ the results reported herein can motivate future studies to evaluate the effectiveness of such an approach for enhancing carboxylic acid HDO as well.

## Supporting Information Available

Adsorption structures, adsorption energetics, reaction energy diagrams, activation energy barriers, and reaction energies (PDF)
