# Supplementary material for: Establishing the Role of Metal, Interface, and Vacancy Sites in Pt/TiO2-Catalyzed Acetic Acid Hydrodeoxygenation
Source: J Phys Chem C Nanomater Interfaces. 2025 Apr 9;129(15):7238–47. doi: 10.1021/acs.jpcc.5c00447 (PMC12010421; doi:10.1021/acs.jpcc.5c00447)
Supplement: Supplementary file 1 — jp5c00447_si_001.pdf [file jp5c00447_si_001.pdf]

Supporting Information

Establishing the Role of Metal, Interface, and Vacancy Sites in Pt/TiO_2-Catalyzed Acetic Acid Hydrodeoxygenation

*Sean A. Tacey and Carrie A. Farberow**

Catalytic Carbon Transformation & Scale Up Center, National Renewable Energy Laboratory, Golden, CO, 80401, USA

*Corresponding author: Carrie.Farberow@nrel.gov

**Supplementary Figures**	**S1-S6**
**Supplementary Tables**	**S7**

Supplementary Figures

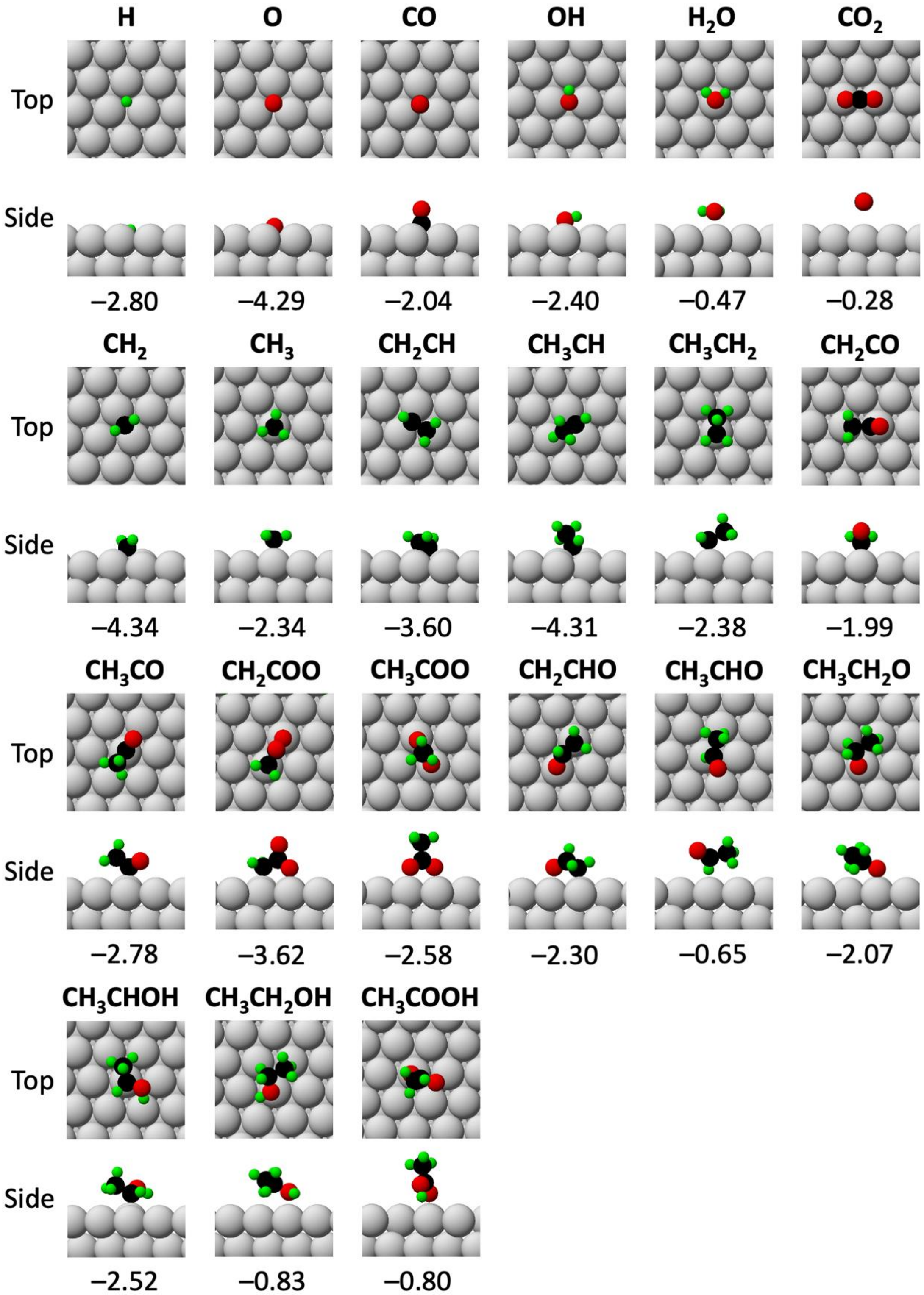

Figure S1. Top and side views of adsorption structures for surface intermediates on Pt(111). Binding energies (in eV) are provided below each snapshot. Atom colors: H – green, C – black, O – red, and Pt – grey.

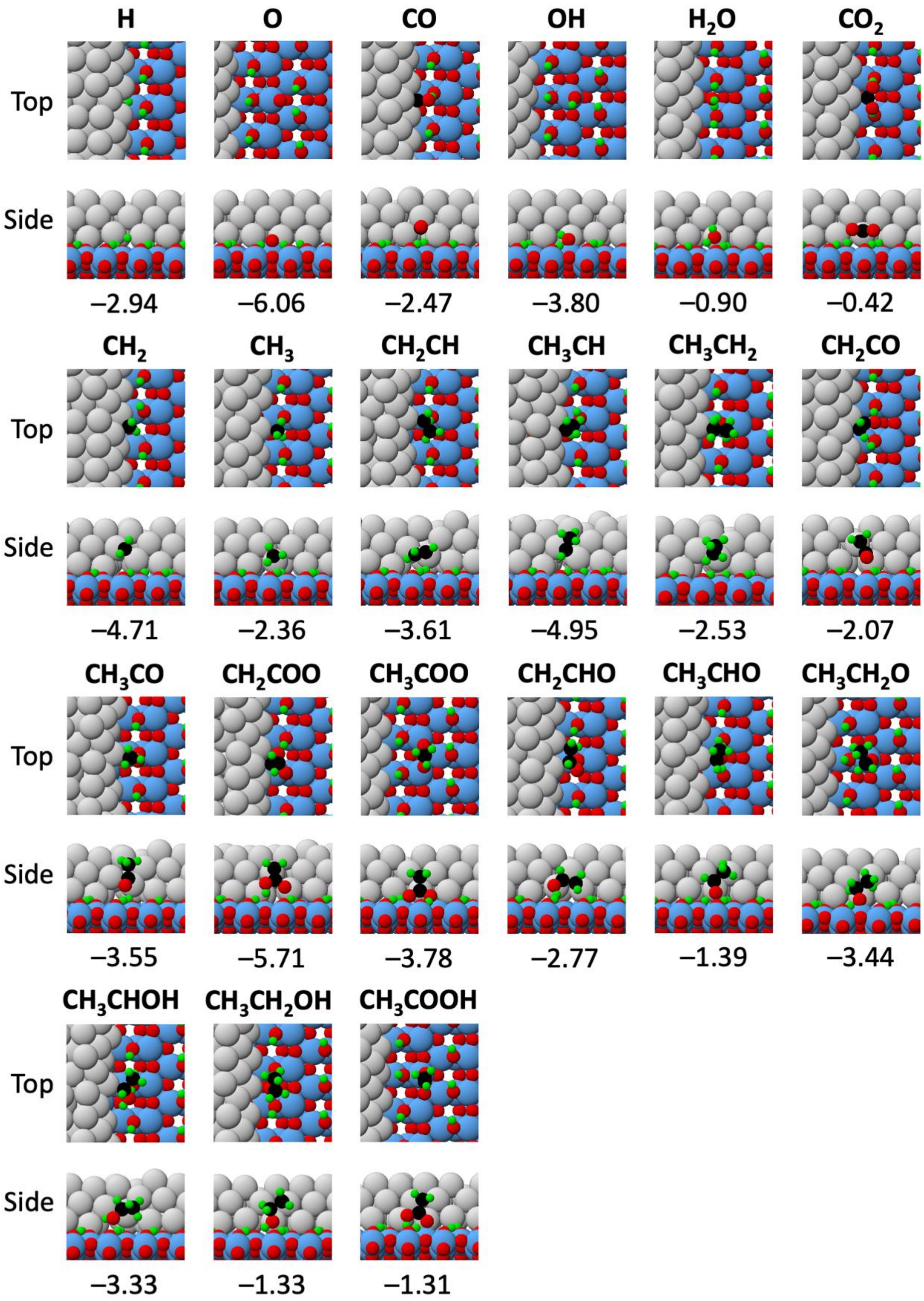

Figure S2. Top and side views of adsorption structures for surface intermediates on Pt_{NW}/OH-TiO_2. Binding energies (in eV) are provided below each snapshot. Atom colors: H – green, C – black, O – red, Pt – grey, and Ti – blue.

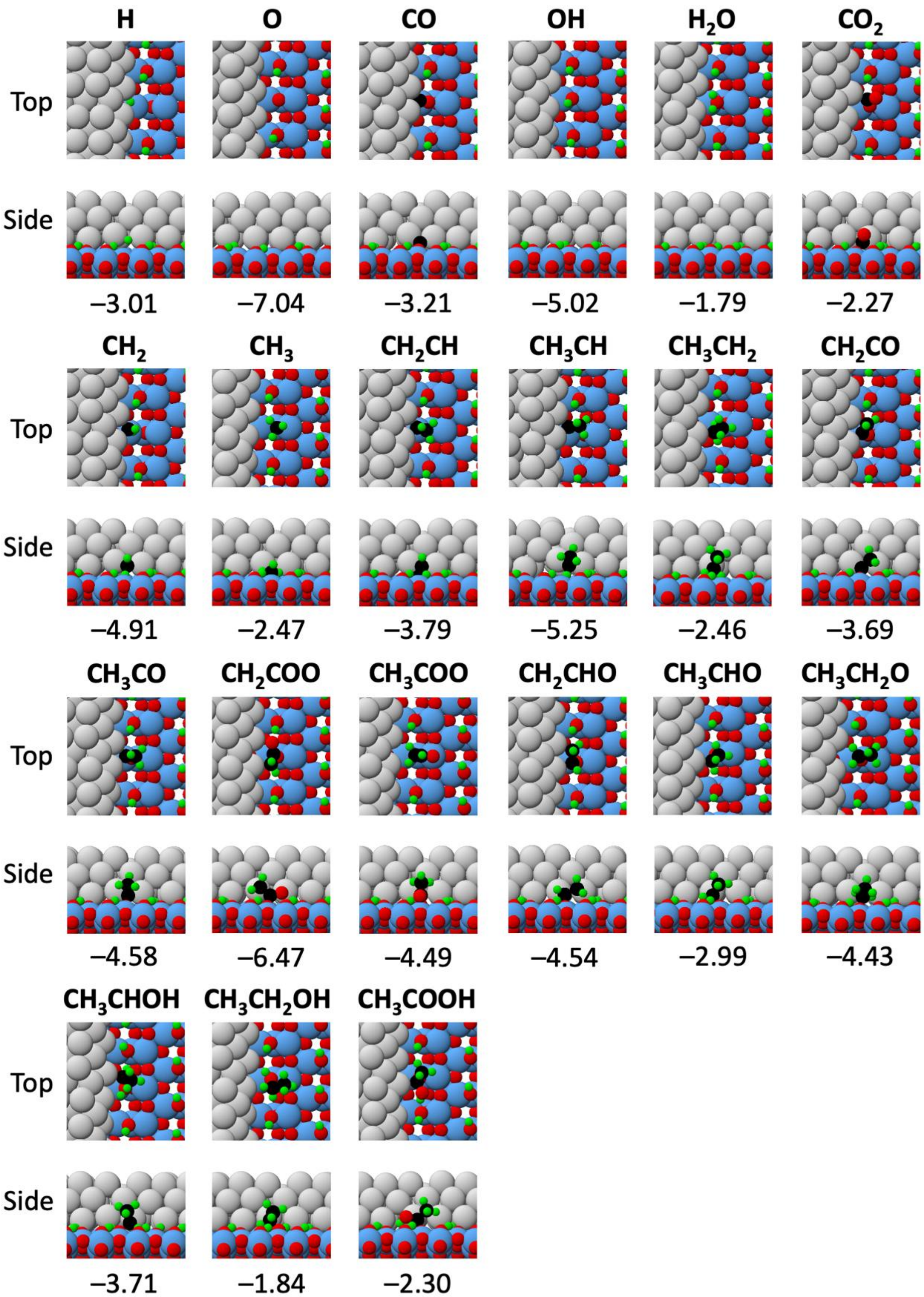

Figure S3. Top and side views of adsorption structures for surface intermediates on Pt_{NW}/OH_v-TiO_2. Binding energies (in eV) are provided below each snapshot. Atom colors: H – green, C – black, O – red, Pt – grey, and Ti – blue.

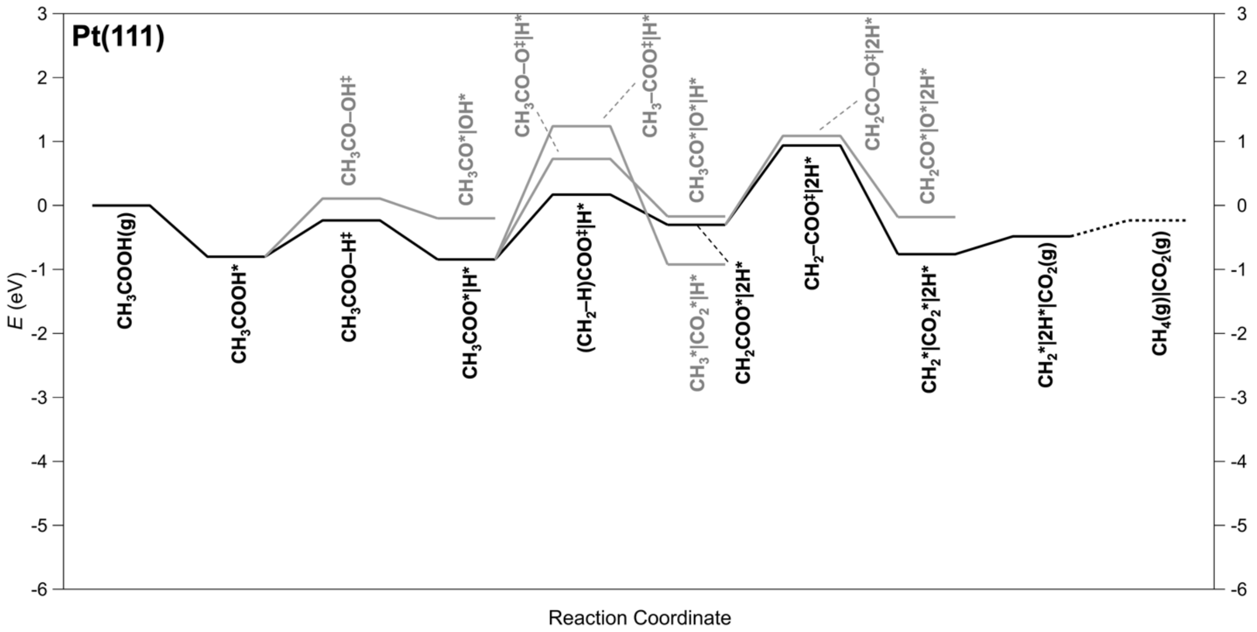

Figure S4. Reaction energy diagram for AA-HDO on Pt(111). Black lines mark the minimum-energy pathway (**Figure 3**), grey lines indicate less-favorable pathways, and dashed lines denote steps not studied. *, |, and ‡ represent adsorbed species, species at infinite separation, and transition states, respectively.

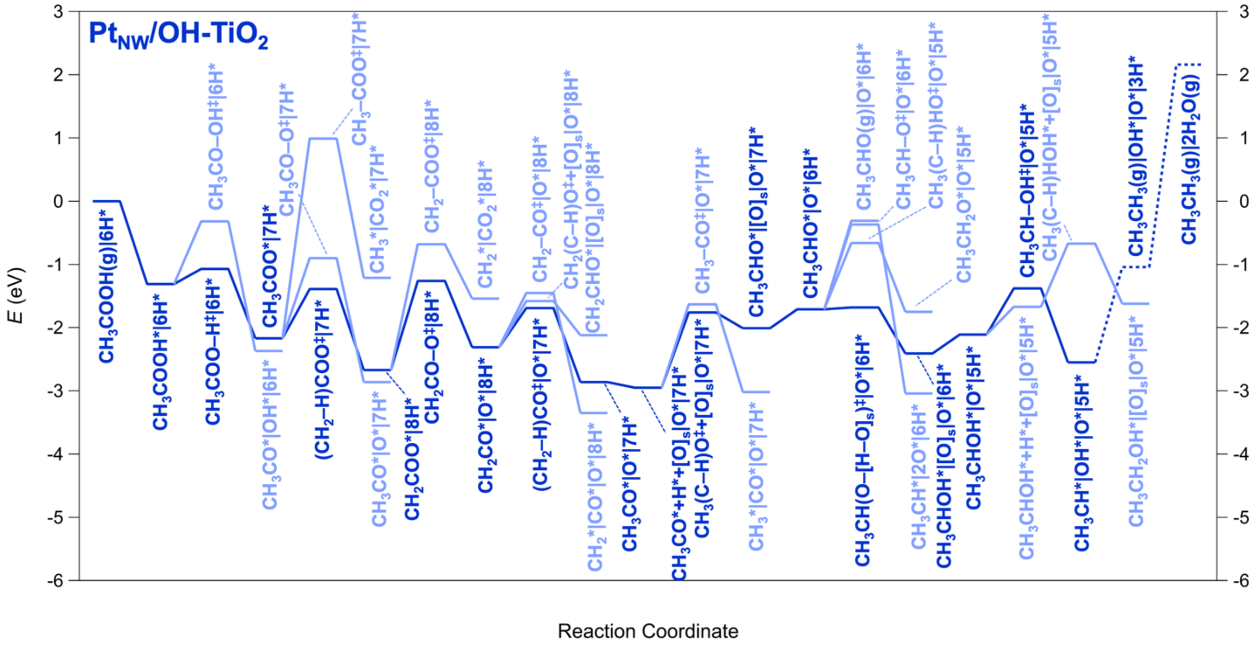

Figure S5. Reaction energy diagram for AA-HDO on $Pt_{NW}/OH-TiO_2$. Dark blue lines mark the minimum-energy pathway (**Figure 3**), light blue lines indicate less-favorable pathways, and dashed lines denote steps not studied. *, |, and ‡ represent adsorbed species, species at infinite separation, and transition states, respectively. $[OH]_s$ and $[O]_s$ indicate surface-OH and surface-O species, respectively.

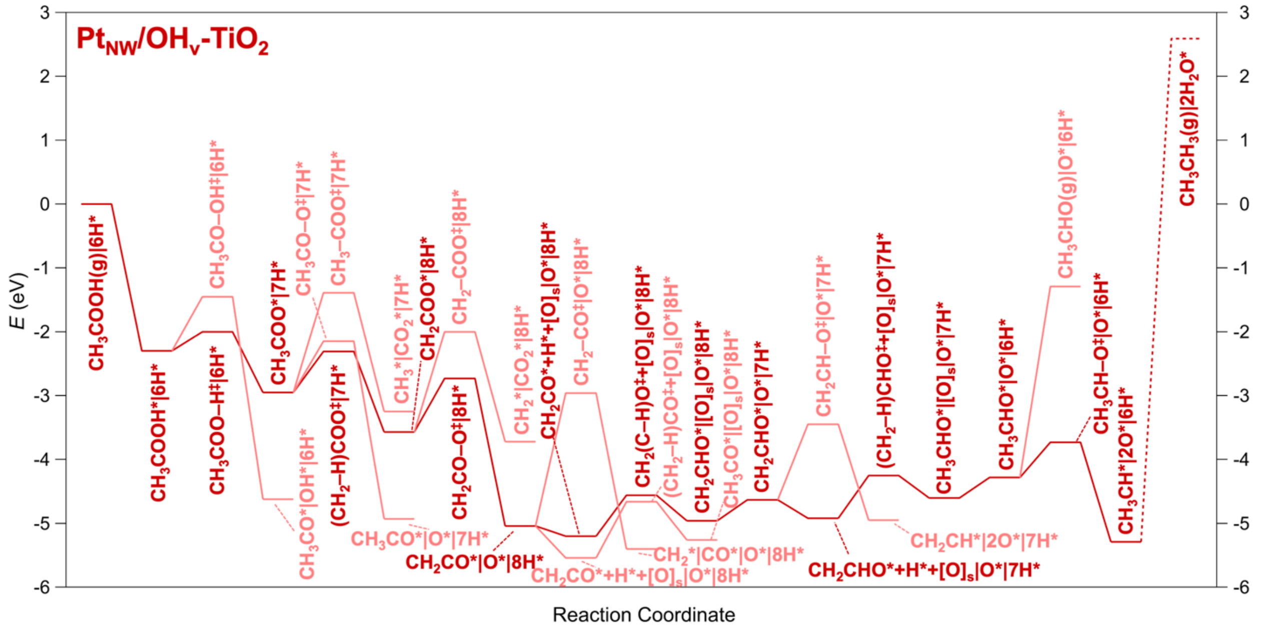

Figure S6. Reaction energy diagram for AA-HDO on Pt_{NW}/OH_v-TiO_2. Dark red lines mark the minimum-energy pathway (**Figure 3**), light red lines indicate less-favorable pathways, and dashed lines denote steps not studied. *, |, and ‡ represent adsorbed species, species at infinite separation, and transition states, respectively. $[O]_s$ indicates surface-O species.

Supplementary Tables

Table S1. Activation energy barriers (E_a, in eV) and reaction energies (ΔE, in eV; in parentheses) for AA-HDO elementary steps on Pt(111), $Pt_{NW}/OH\text{-}TiO_2$, and $Pt_{NW}/OH_v\text{-}TiO_2$. * denotes an adsorbed species or vacant site.

	E_a (ΔE) (eV)		
Elementary step	Pt(111)	$Pt_{NW}/OH\text{-}TiO_2$	$Pt_{NW}/OH_v\text{-}TiO_2$
C-C bond-breaking			
$CH_3COO^* + {}^* \rightarrow CH_3^* + CO_2^*$	2.08 (–0.08)	3.16 (0.96)	1.58 (–0.29)
$CH_2COO^* + {}^* \rightarrow CH_2^* + CO_2^*$	1.25 (–0.46)	1.99 (1.13)	1.57 (–0.15)
$CH_3CO + {}^* \rightarrow CH_3^* + CO^*$	1.34 (–0.49)	1.23 (–0.17)	1.34 (0.00)
$CH_2CO + {}^* \rightarrow CH_2^* + CO^*$	1.07 (–0.32)	0.86 (–1.05)	2.07 (–0.36)
C-O bond-breaking			
$CH_3COOH^* + {}^* \rightarrow CH_3CO^* + OH^*$	0.91 (0.60)	0.98 (–1.06)	0.85 (–2.32)
$CH_3COO^* + {}^* \rightarrow CH_3CO^* + O^*$	1.57 (0.67)	1.27 (–0.69)	0.82 (–1.98)
$CH_2COO^* + {}^* \rightarrow CH_2CO^* + O^*$	1.40 (0.12)	1.41 (0.36)	0.84 (–1.47)
$CH_3CHO^* + {}^* \rightarrow CH_3CH^* + O^*$	1.41 (0.35)	1.34 (–1.33)	0.55 (–1.02)
$CH_2CHO^* + {}^* \rightarrow CH_2CH^* + O^*$	1.68 (0.38)	1.09 (–0.94)	1.18 (–0.32)
$CH_3CH_2O^* + {}^* \rightarrow CH_3CH_2^* + O^*$	1.46 (–0.17)	1.90 (–0.74)	1.29 (–0.65)
$CH_3CHOH + {}^* \rightarrow CH_3CH^* + OH^*$	1.27 (0.79)	0.72 (–0.45)	0.19 (–1.58)
$CH_3CH_2OH + {}^* \rightarrow CH_3CH_2^* + OH^*$	1.79 (0.37)	0.92 (–0.69)	0.78 (–1.32)
Dehydrogenation			
$CH_3COOH^* + {}^* \rightarrow CH_3COO^* + H^*$	0.57 (–0.04)	0.24 (–0.86)	0.30 (–0.66)
$CH_3COO^* + {}^* \rightarrow CH_2COO^* + H^*$	1.01 (+0.54)	0.78 (–0.50)	0.65 (–0.62)
$CH_3CO^* + {}^* \rightarrow CH_2CO^* + H^*$	0.97 (–0.01)	1.41 (0.55)	1.24 (–0.11)
Hydrogenation			
$CH_3CHOH^* + H^* \rightarrow CH_3CH_2OH^* + {}^*$	1.19 (0.35)	1.00 (+0.79)	0.82 (0.74)
$CH_3CH_2O^* + H^* \rightarrow CH_3CH_2OH^* + {}^*$	0.24 (–0.57)	0.60 (+0.44)	1.05 (0.99)
$CH_3CHO^* + H^* \rightarrow CH_3CHOH^* + {}^*$	0.23 (–0.46)	0.02 (–0.40)	0.88 (0.88)
$CH_3CHO^* + H^* \rightarrow CH_3CH_2O^* + {}^*$	0.58 (0.45)	1.04 (–0.05)	0.94 (0.63)
$CH_3CO^* + H^* \rightarrow CH_3CHO^* + {}^*$	1.18 (0.99)	1.19 (1.15)	0.66 (0.66)
$CH_2CHO^* + H^* \rightarrow CH_3CHO^* + {}^*$	1.00 (0.24)	0.31 (0.11)	0.67 (0.36)
$CH_2CO^* + H^* \rightarrow CH_3CO^* + {}^*$	1.45 (0.01)	0.61 (–0.55)	0.87 (0.11)
$CH_2CO^* + H^* \rightarrow CH_2CHO^* + {}^*$	0.98 (0.75)	0.73 (0.50)	0.63 (0.41)
